# Thioredoxin1 Downregulates Oxidized Low-Density Lipoprotein-Induced Adhesion Molecule Expression via Smad3 Protein

**DOI:** 10.1371/journal.pone.0076226

**Published:** 2013-09-23

**Authors:** Beidong Chen, Wendong Wang, Tao Shen, Ruomei Qi

**Affiliations:** Key Laboratory of Geriatrics, Beijing Institute of Geriatrics & Beijing Hospital, Ministry of Health, Beijing, China; University of Milan, Italy

## Abstract

Atherosclerosis is a chronic inflammation disease that is initiated by endothelial cell injury. Oxidized low-density lipoprotein (ox-LDL) is directly associated with chronic vascular inflammation. To understand whether thioredoxin1 (Trx1) participates in an antiinflammatory defense mechanism in atherosclerosis, we investigated the effect of Trx1 on the expression of two adhesion molecules, vascular cell adhesion molecule-1 (VCAM-1) and intercellular adhesion molecule-1 (ICAM-1), in human umbilical vein endothelial cells (HUVECs). Thioredoxin1 and dominant-negative mutant thioredoxin1 (TD) were transiently overexpressed using adenovirus vector gene transfer. Our data showed that Trx1 overexpression suppressed ox-LDL-induced adhesion molecule expression in HUVECs. The overexpression of Trx1 promoted ox-LDL-induced Smad3 phosphorylation and nuclear translocation. A co-immunoprecipitation assay indicated that Smad3 continued to interact with Trx1 with or without ox-LDL stimulation. These results suggest that Trx1 inherently suppresses VCAM-1 and ICAM-1 expression in vascular endothelia and may prevent the initiation of atherosclerosis by attenuating adhesion molecule expression. The enhancement of Smad3 phosphorylation and nuclear expression appears to be primarily responsible for the Trx1-induced downregulation of adhesion molecules.

## Introduction

Oxidized low-density lipoprotein (ox-LDL) is well known to play a crucial role in the initiation and progression of atherosclerosis, which can be considered an inflammatory disease [1]. Modified LDL can induce endothelial cell activation and the expression of adhesion molecules that facilitate the firm adhesion and activation of leukocytes and platelets, thereby enhancing inflammatory processes that underlie atherosclerosis [2]. Smad transcription factors are specific downstream mediators of the transforming growth factor β (TGF-β) signaling pathway. TGF-β is a multifunctional cytokine that regulates cell proliferation, differentiation, apoptosis, and extracellular matrix accumulation [3]. Smad3 belongs to receptor-regulated Smads (R-Smads) and can be activated by TGF-β and activin receptors. TGF-β has an antiatherogenic effect, in which it prevents the ox-LDL-induced expression of adhesion molecules [4] and contributes to plaque stabilization [5]. Additionally, the disruption of TGF-β signaling in T cells accelerates atherosclerosis in apolipoprotein E knockout mice [6]. In vascular cells, cholesterol suppressed TGF-β signaling by increasing lipid rafts and the caveolae accumulation of TGF-β receptors [7]. Although the TGF-β/Smad pathway has been shown to have protective, antiinflammatory effects on cells that are important to atherosclerotic lesion formation [8], remaining unclear is how Smad3 contributes to ox-LDL stimulation in human umbilical vein endothelial cells (HUVECs).

Thioredoxin (Trx), a small, ubiquitous thiol protein, is one of the most important regulators of redox balance. It reduces oxidized cysteine groups in proteins by interacting with its redox-active center Cys-Gly-Pro-Cys, which in turn can be reduced by Trx reductase (TrxR) and NADPH [9]. Three isoforms of human Trx that are encoded by separate genes have been identified. Trx1 is a 104-amino-acid protein that is found in both the cytoplasm and nuclei of cells [10]. In contrast, Trx2 is a 166-amino-acid protein that contains a 60-amino-acid NH2-terminal translocation sequence that directs it to the mitochondria [11]. The third isoform, SpTrx, is a variant that is highly expressed in spermatozoa [12]. Unless otherwise indicated, Trx refers to Trx1 in the present work. The processes influenced by Trx include the control of cellular redox balance [10], [13], promotion of cell growth [14], inhibition of apoptosis [15], and modulation of inflammation [16]. Our previous work showed that Trx downregulated monocyte chemoattractant protein-1 secretion and expression in a human endothelial cell line by suppressing the nuclear translocation of activator protein-1 (AP-1) and redox factor-1 [17]. These studies indicate that Trx may play a role in the pathogenesis of atherosclerosis. However, to date, the participation of Trx in the protection against vascular endothelium atherosclerosis remains unclear. Although both Trx and Smad3 have antiinflammatory effects on cells [8], [18] that are important to atherosclerotic lesion formation, no studies of which we are aware have reported the relationship between these two proteins. We used HUVECs to establish cells that overexpressed Trx or dominant-negative Trx and investigated the effects of Trx on Smad3 and adhesion protein expression in HUVECs.

## Materials and Methods

### Ethics statement

According to the Declaration of Helsinki, umbilical cords were donated by cesarean section patients, from whom we received written informed consent. The study was approved by the ethics committee of the Beijing Institute of Geriatrics, Ministry of Health (approval no. 2006-05).

### Reagents

SIS3(S0447), 5,5′-dithiobis (2-nitrobenzoic acid; DTNB), thioredoxin reductase (TrxR) from rat liver, phenylmethanesulfonyl fluoride (PMSF), Protease Inhibitor Cocktail, and type I collagenase were purchased from Sigma-Aldrich (St. Louis, MO, USA). 2′,7′-Dichlorodihydrofluorescein diacetate (DCFH-DA) was obtained from the Beyotime Institute of Biotechnology (Shanghai, China). Anti-rabbit biotinylated antibody was obtained from Beijing Zhongshan Golden Bridge Biotechnology Co., Ltd (Beijing, China). The Trx1 expression vector pcDNA3-Trx1 and redox-inactive dominant-negative mutant Trx (TD; Cys32→Ser32/Cys35→Ser35[C32S/C35S]) expression vector pcDNA3-TD were kindly provided by Dr. J. Yodoi (Institute for Virus Research, Kyoto University, Kyoto, Japan). NE-PER nuclear and cytoplasmic extraction reagents and the BCA assay kit were purchased from Pierce (Rockford, IL, USA). All of the other reagents were of analytical grade.

### Preparation and culture of HUVECs

Freshly isolated umbilical cords were obtained from healthy donors. Primary HUVECs were isolated from the umbilical vein using type I collagenase and then cultured in Dulbecco Modified Eagle Medium supplemented with 20% fetal bovine serum, endothelial cell growth factor, 100 U/ml penicillin, 100 U/ml streptomycin, and 1% glutamine in a humidified incubator at 37°C and 5% CO_2_. HUVECs at passages 2–4 were used in the present study.

### Construction of Trx and dominant-negative mutant thioredoxin (TD) adenovirus

The ViraPower Adenoviral Gateway Expression system from Invitrogen (Carlsbad, CA, USA) was used to construct green fluorescent protein (GFP), Trx, and TD adenovirus expression vectors. The entry vector, pENTR/D-TOPO, was kindly provided by Dr. Jianping Cai (Beijing Institute of Geriatrics). The DNA restriction enzymes KpnI and XbaI were purchased from TaKaRa Bio Company (Otsu, Shiga, Japan). T4 DNA Ligase and PacI were purchased from Promega (Madison, WI, USA) and New England BioLabs (Ipswich, MA), respectively. We constructed adenovirus expression vectors according to the manufacturer's protocols. Briefly, target fragments were digested from a pcDNA3.0 vector and inserted into the Entry vectors. The gateway technique was used to recombine and generate adenovirus expression vectors. After the identification of the adenovirus expression vectors, these plasmids were purified and digested using PacI. The 293A cell line (provided by the ViraPower Adenoviral Gateway Expression Kit, Invitrogen) was used to package the adenovirus. After 8–10 days of transfection, the viruses were harvested. HUVECs were infected with an adenovirus that contained GFP (Ad-GFP) as a control group, Trx (Ad-Trx), and TD (Ad-TD) for 60 h to overexpress GFP, Trx, and TD in HUVECs, respectively.

### Knockdown of Trx1 by siRNA

The sequence of the small-interfering RNA (siRNA) used against Trx1 was 5′-AUGACUGUCAGGAUGUUGCdTdT-3′ [19]. The scramble oligonucleotide 5′-UUCUCCGAACGUGUCACGUTT-3′ was used as a negative control (NC). The cells were seeded in six-well plates and cultured overnight and transfected with 50 nM siRNA or NC oligonucleotide, respectively, using Effectene Transfection Reagent (Qiagen), according to the manufacturer's instructions. All of the experiments with Trx1-knockdown cells were performed 60 h after transfection.

### Assay of Trx activity in HUVECs

The insulin disulfide reduction assay was essentially performed as described elsewhere [20], with a slight modification. Transiently transfected cells were lysed in lysis buffer (20 mM HEPES [pH 7.9], 100 mM KCl, 300 mM NaCl, 10 mM ethylenediaminetetraacetic acid [EDTA], and 0.1% Nonidet P-40 plus protease inhibitors). Cell extracts (20 µg) were preincubated at 37°C for 20 min with 2 µl of dithiothreitol (DTT) activation buffer that consisted of 50 mM HEPES (pH 7.6), 1 mM EDTA, 1 mg/ml bovine serum albumin (BSA), and 2 mM DTT in a total volume of 70 µl to reduce Trx. The final concentration of DTT was 57 µM. Afterward, 40 µl of reaction mixture (256 mM HEPES [pH 7.6], 10 mM EDTA, 2.05 mg/ml nicotinamide adenine dinucleotide phosphate [NADPH], and 6.4 mg/ml insulin) was added. The reaction began with the addition of 10 µl of rat TrxR (100 A412 U/ml), and incubation continued for 20 min at 37°C. The reaction was stopped by the addition of 0.5 ml of 6 M guanidine-HCl and 1 mM DTNB (3-carboxy-4-nitrophenyl disulfide), and absorbance was measured at 412 nm.

### Isolation and oxidation of LDL

Human LDL was isolated from the plasma of healthy donors by sequential ultracentrifugation using a previously described method [21]. The concentration of LDL protein was determined using a UV-160A ultraviolet-visible spectrum spectrophotometer (Shimadzu, Kyoto, Japan). For oxidation, LDL (3–5 mg/ml) was incubated with 5 µM CuSO_4_ as the oxidant for 12–16 h at room temperature and quenched by the addition of 2 mM EDTA. The ox-LDL preparation was sterilized through sterile 0.22-µm Millex syringe-driven filters. The LDL oxidation level (0.1 µM malondialdehyde (MDA)/mg protein) was determined by the thiobarbituric acid reactive substance (TBARS) assay.

### Western blot analysis

Cell lysates (15–30 µg protein) were separated by 12% sodium dodecyl sulfate-polyacrylamide gel electrophoresis (SDS-PAGE), transferred to a polyvinylidene fluoride (PVDF) membrane (Millipore, Billerica, MA, USA), blocked with 5% nonfat dry milk, and probed with antibodies at 4°C overnight. The blots were incubated with horseradish peroxidase (HRP)-conjugated anti-immunoglobulin G (IgG), followed by electrochemiluminescence (ECL) detection (Millipore, Billerica, MA, USA). Antibodies against Trx1 (FL-105) and β-actin (C4) were purchased from Santa Cruz Biotechnology (Santa Cruz, CA, USA). Anti-Smad3 (C67H9) rabbit monoclonal antibody (mAb), anti-phosphorylated Smad3 (pSmad3; Ser423/425; C25A9) rabbit mAb, and anti-histone H3 antibody were purchased from Cell Signaling Technology (Danvers, MA, USA).

### Monocyte-endothelial cell adhesion assay

U937 monocytes (1×10^7^ cells/ml) were incubated with 10 µM 2′,7′-bis-(2-carboxyethyl)-5-(and-6)-carboxy-fluorescein acetoxymethyl ester (BCECF-AM; Beyotime Institute of Biotechnology, Shanghai, China) for 30 min at 37°C in RPMI-1640 medium. HUVECs (5×10^5^ cells/well) were seeded in six-well plates and stimulated with 100 µg/ml ox-LDL for 6 h. The HUVECs were then washed with PBS three times to remove ox-LDL. The fluorescent-labeled U937 monocytes were added to the stimulated HUVECs and incubated for a further 2 h. After washing out the unbound U937 three times, monocyte adhesion was measured by detecting the fluorescent intensity using a Fluoroskan (Thermo Scientific). Wells that contained HUVECs alone were used as blanks.

### Immunoprecipitation and immunoblotting

Lysates (500 µg protein) were immunoprecipitated with 2 µg of Trx antibody overnight at 4°C. After incubation with protein A and protein G-sepharose (GE Healthcare, Munich, Germany) for 2 h at 4°C, the resulting beads were washed and boiled in SDS-PAGE sample buffer, and the proteins were resolved by SDS-PAGE. Immunoblotting was performed with antibodies directed against Smad3 and pSmad3. To avoid the influence of the heavy chain in the homology antibody, a specific secondary antibody to rabbit IgG light chain (HRP) was used, and the proteins were then detected using an ECL kit. Mouse monoclonal (SB62a) secondary antibody to rabbit IgG light chain (HRP) was purchased from Abcam (Boston, MA, USA).

### Detection of reactive oxygen species generation in cells

DCFH-DA was used to detect intracellular reactive oxygen species (ROS) generation (49). Briefly, Ad-GFP, Ad-Trx, and Ad-TD cells were cultured overnight and then loaded with 10 µM DCFH-DA for 30 min. The ROS indicator in the medium was then washed off. After three additional washes, the cells were digested with trypsin. The cells were then harvested and determined at an excitation wavelength of 480 nm and emission wavelength of 520 nm on an F-4500 Fluorescence Spectrophotometer (Hitachi).

### Data analysis

The data are expressed as mean ±SEM. Statistical comparisons were made using one-way analysis of variance (ANOVA) followed by the Bonferroni test for multiple-group comparisons. Values of *p*<0.05 were considered statistically significant.

## Results

### Thioredoxin downregulated VCAM-1 and ICAM-1 expression in HUVECs

**Figure 1:**
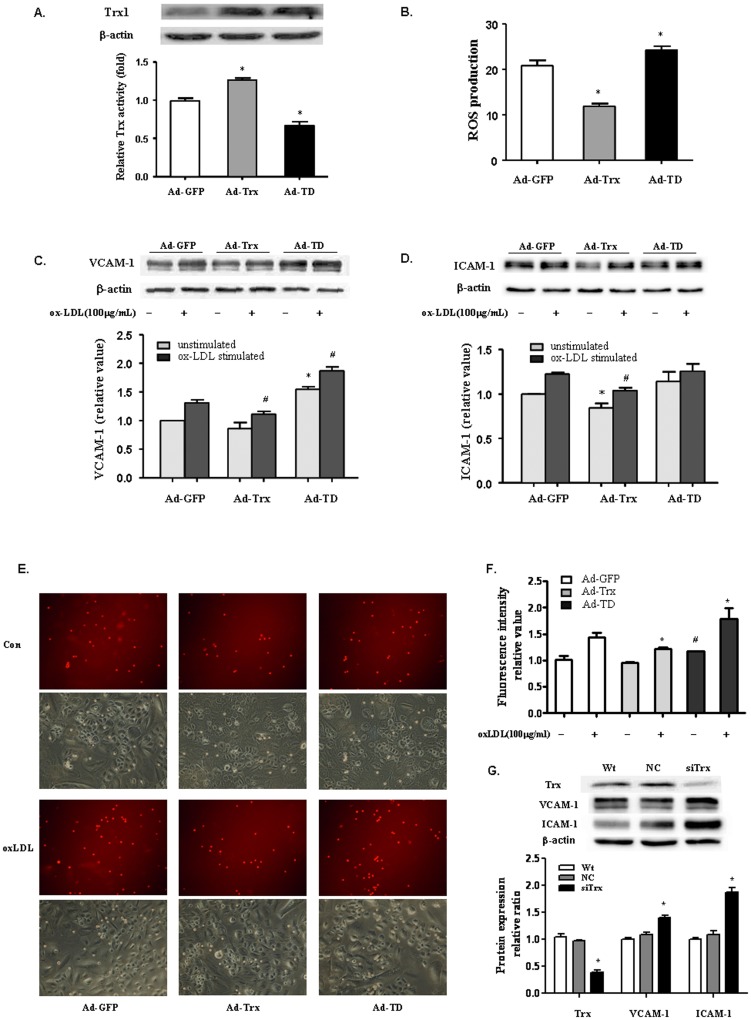
Thioredoxin downregulates VCAM-1 and ICAM-1 expression in HUVECs. (**A**) *Top*, Immunoblot of Trx1 in HUVECs infected by GFP adenovirus (Ad-GFP), Trx adenovirus (Ad-Trx), and TD adenovirus (Ad-TD). *Bottom*, Trx1 activity in Ad-GFP, Ad-Trx, and Ad-TD cells, determined by insulin reduction-based assay. (**B**) Reactive oxygen species production in HUVECs. After 2 h stimulation of ox-LDL (100 µg/ml), ROS production was analyzed by measuring the mean fluorescence intensity using flow cytometry. (**C**, **D**) Immunoblot of VCAM-1 and ICAM-1 in Ad-GFP, Ad-Trx, and Ad-TD cells under basal conditions and after 4 h ox-LDL (100 µg/ml) stimulation. Relative VCAM-1 and ICAM-1 expression was determined by densitometric analysis. In all of the histograms, each value represents the mean ±SEM (*n* = 3 independent measurements). *p<0.05, compared with unstimulated Ad-GFP cells; ^#^ *p*<0.05, compared with ox-LDL-stimulated Ad-GFP cells. (**E**) U937 monocyte adhesion assay in ox-LDL-stimulated Ad-GFP, Ad-Trx, and Ad-TD cells. U937 cells that adhered to HUVECs were observed under a fluorescent microscope at 200× magnification. (**F**) The intensity of fluorescence-labeled adherent U937 monocytes was measured with a fluorometer (excitation wavelength, 485 nm; emission wavelength, 530 nm). The intensity was normalized to that of control cells in each group. The data are expressed as mean ±SEMs (*n* = 5). *p<0.05, compared with ox-LDL-treated Ad-GFP group; ^#^ *p*<0.05, compared with unstimulated Ad-GFP group. (**G**) *Top*, Immunoblot of Trx1, VCAM-1, and ICAM-1 in wildtype HUVECs (Wt) and HUVECs transfected by negative control siRNA (NC) or Trx siRNA (si-Trx). *Bottom*, Relative Trx, VCAM-1, and ICAM-1 expression was determined by densitometric analysis. In all of the histograms, each value represents the mean ±SEM (*n* = 3 independent measurements). *p<0.05, compared with Wt cell.

To determine whether Trx plays a role in the regulation of VCAM-1 and ICAM-1 expression in HUVECs, the expression of these two adhesion molecules was analyzed in cells that overexpressed Trx and dominant-negative Trx. The immunoblotting analysis showed that the protein levels of Trx in the Ad-Trx and Ad-TD groups were increased compared with the Ad-GFP control group. As expected, the insulin reduction-based assays showed that Trx activity increased in the Ad-Trx group but decreased in the Ad-TD group compared with the Ad-GFP group (Fig. 1A). Functional Trx eliminated intracellular ROS by providing electrons to the peroxiredoxin-catalyzed reduction of ROS; therefore, the amount of intracellular ROS could reflect Trx activity. As shown in Fig. 1B, ROS generation was inhibited in the Ad-Trx group. In contrast, enhanced ROS production in HUVECs was found in the TD group. These results indicate that Ad-Trx overexpressed functional Trx, whereas Ad-TD only enhanced redox-dysfunctional Trx. To determine whether Trx affects the expression of ICAM-1 and VCAM-1 in HUVECs, adenovirus-infected HUVECs were treated with or without ox-LDL for 6 h, and protein levels were detected by Western blot. As shown in Fig. 1C and D, Trx overexpression inhibited ICAM-1 and VCAM-1 expression under both basal and ox-LDL-stimulated conditions, whereas TD overexpression did not have this protective effect and even significantly enhanced VCAM-1 expression. To verify the functional effects of Trx in HUVECs, a monocyte-endothelial cell adhesion assay was performed. As shown in Fig. 1E and F, consistent with the Western blot results, Trx overexpression inhibited cell adhesion to ox-LDL-stimulated HUVECs, whereas TD enhanced adhesion. Additionally, the effect of Trx on VCAM-1 and ICAM-1 expression was investigated in cells that had their endogenous Trx1 knocked down by siRNA (siTrx). As shown in Fig. 1G, VCAM-1 and ICAM-1 expression was significantly enhanced in siTrx cells under basal conditions.

### Native LDL failed to increase adhesion molecule expression in HUVECs

**Figure 2:**
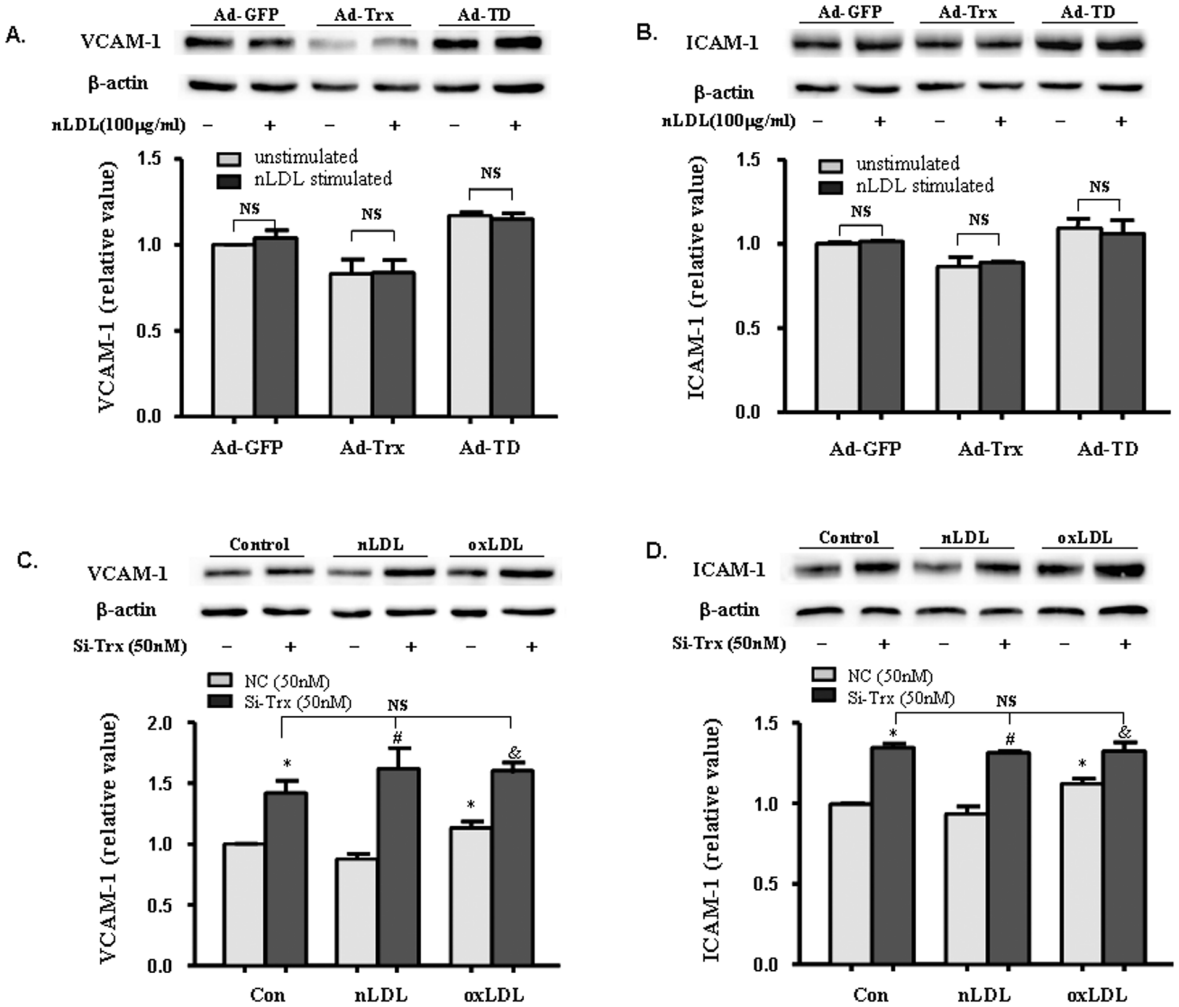
Effect of native LDL (nLDL) on the expression of VCAM-1 and ICAM-1 in Trx-overexpressing and -knock-down HUVECs. (**A, B**) Immunoblot of VCAM-1 and ICAM-1 in Ad-GFP, Ad-Trx, and Ad-TD cells under basal conditions and after 4 h nLDL (100 µg/ml) stimulation. (**C, D**) Immunoblot of VCAM-1 and ICAM-1 in NC and si-Trx cells under basal conditions and after 4 h nLDL (100 µg/ml) or ox-LDL (100 µg/ml) stimulation. Relative VCAM-1 and ICAM-1 expression was determined by densitometric analysis. In all of the histograms, each value represents the mean ±SEM (*n* = 3 independent measurements). *p<0.05, compared with unstimulated NC cells; ^#^ *p*<0.05, compared with nLDL-stimulated NC cells; ^&^ *p*<0.05, compared with ox-LDL-stimulated NC cells.

Native LDL (nLDL) is a critical control of ox-LDL. We detected VCAM-1 and ICAM-1 expression in nLDL-stimulated Ad-GFP, Ad-Trx, and Ad-TD cells. As shown in Fig. 2A, nLDL did not significantly enhance adhesion molecule expression in the three HUVECs groups. VCAM-1 and ICAM-1 expression was also detected in nLDL- and ox-LDL-stimulated Trx knock-down cells. Unexpectedly, Trx knock-down greatly increased the expression of adhesion molecules to such an extent that nLDL and ox-LDL failed to further enhance their expression.

### Thioredoxin induced Smad3 phosphorylation in ox-LDL-treated HUVECs

**Figure 3:**
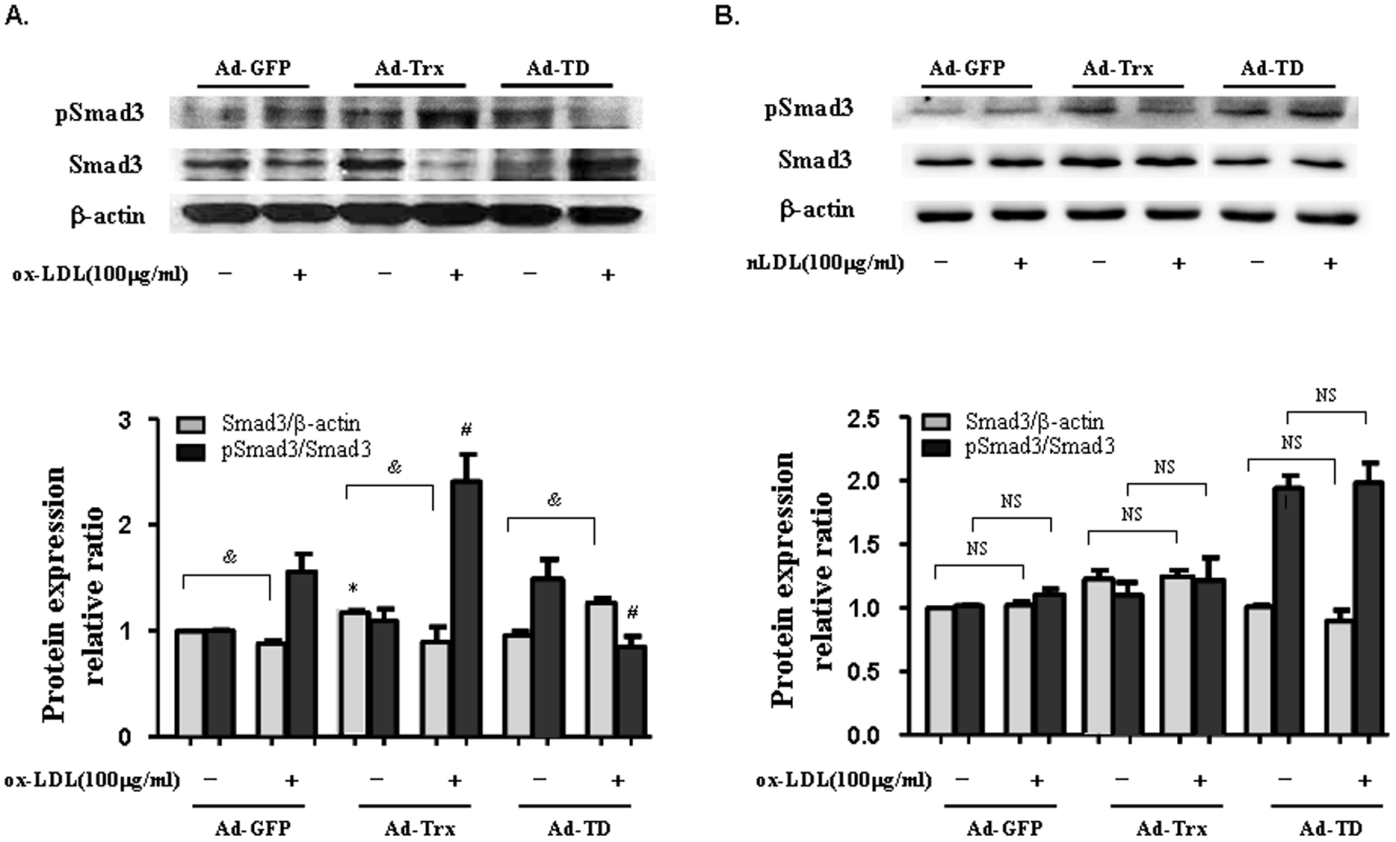
Effect of Trx on the expression of pSmad3 and Smad3 in HUVECs stimulated with ox-LDL and nLDL. (**A**) Immunoblot of pSmad3 and Smad3 expression in ox-LDL-stimulated Ad-GFP, Ad-Trx, and Ad-TD HUVECs. (**B**) Immunoblot of pSmad3 and Smad3 expression in nLDL-treated Ad-GFP, Ad-Trx, and Ad-TD HUVECs. Relative pSmad3 and Smad3 content was determined by densitometric analysis. The data from three separate experiments are expressed as mean ±SEM. *p<0.05, compared with unstimulated Ad-GFP cells (Smad3/β-actin); ^#^ *p*<0.05, compared with ox-LDL-stimulated Ad-GFP cells (pSmad3/Smad3); ^&^ *p*<0.05, unstimulated cells compared with ox-LDL stimulated cells (Smad3/β-actin).

To ascertain whether the antiinflammatory effect of Trx interacts with the Smad3 pathway, the expression levels of pSmad3 and Smad3 were detected in the three groups of cells under basal and ox-LDL-stimulated conditions. As shown in Fig. 3A, ox-LDL stimulation decreased Smad3 expression in the Ad-GFP and Ad-Trx groups but had an opposite effect in the Ad-TD group. Trx overexpression further enhanced Smad3 phosphorylation and TD overexpression decreased Smad3 phosphorylation compared with the Ad-GFP control group after ox-LDL stimulation in HUVECs. Additionally, nLDL was used as a control for ox-LDL. As shown in Fig. 3B, no significant difference was observed compared with the unstimulated groups after nLDL stimulation in HUVECs. These results indicate that Trx plays an important regulatory role in Smad3 expression and phosphorylation.

### SIS3, a specific inhibitor of Smad3 phosphorylation, reversed the Trx-induced inhibition of ICAM-1 and VCAM-1 expression

**Figure 4:**
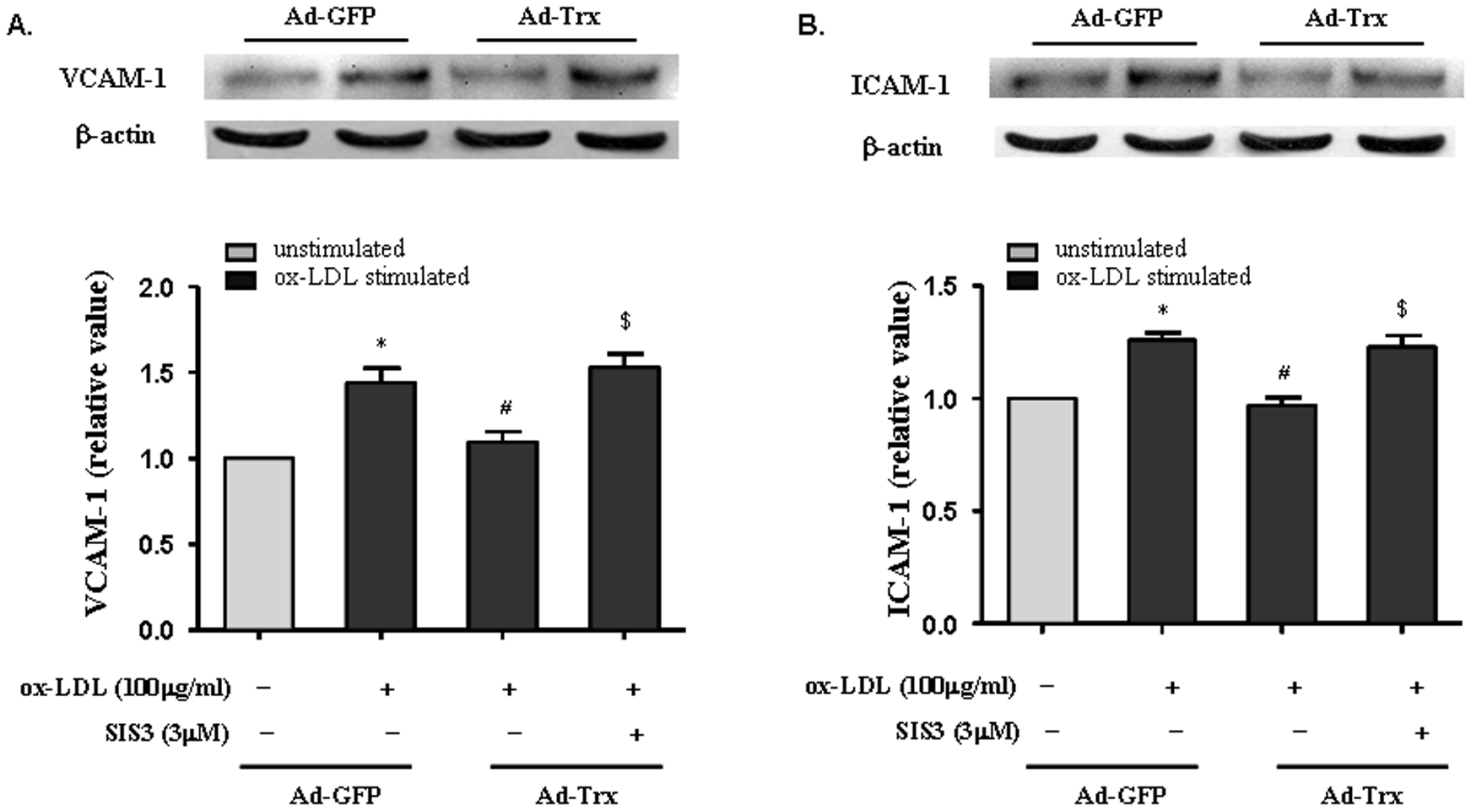
Role of SIS3 in VCAM-1 and ICAM-1 expression in HUVECs stimulated with ox-LDL. (**A**) Overexpression of Trx suppressed ox-LDL-induced VCAM-1 expression. The pretreatment of Ad-Trx cells with SIS3 for 1 h completely reversed the inhibitory effect of Trx on VCAM-1 expression. (**B**) The overexpression of Trx inhibited ox-LDL-induced ICAM-1 expression. The pretreatment of Ad-Trx cells with SIS3 reversed the inhibitory effect of Trx on ICAM-1 expression. The relative expression of VCAM-1 and ICAM-1 was determined by densitometric analysis. The data from three separate experiments are expressed as mean ±SEM. *p<0.05, compared with unstimulated Ad-GFP cells; ^#^ *p*<0.05, compared with ox-LDL-stimulated Ad-GFP cells; ^$^ *p*<0.05, compared with ox-LDL-stimulated Ad-Trx cells.

The TGF-β/Smad pathway contributes to antiatherosclerotic effects [8], but remaining unclear is the role of Smad3 in HUVECs under ox-LDL stimulation conditions. SIS3, a specific inhibitor of Smad3, attenuated the TGF-β1-induced phosphorylation of Smad3 and interaction between Smad3 and Smad4 [22]. ICAM-1 and VCAM-1 expression was analyzed by pretreating the cells with SIS3 for 1 h, followed by 6 h ox-LDL stimulation. As shown in Fig. 4, SIS3 reversed the Trx-induced inhibition of ICAM-1 and VCAM-1 expression in the Ad-GFP and Ad-Trx groups after ox-LDL stimulation. These data indicate that the Smad3 pathway may be involved in the Trx-induced inhibition of adhesion molecules in HUVECs.

### Trx had a constitutive interaction with Smad3 and pSmad3 in HUVECs

**Figure 5:**
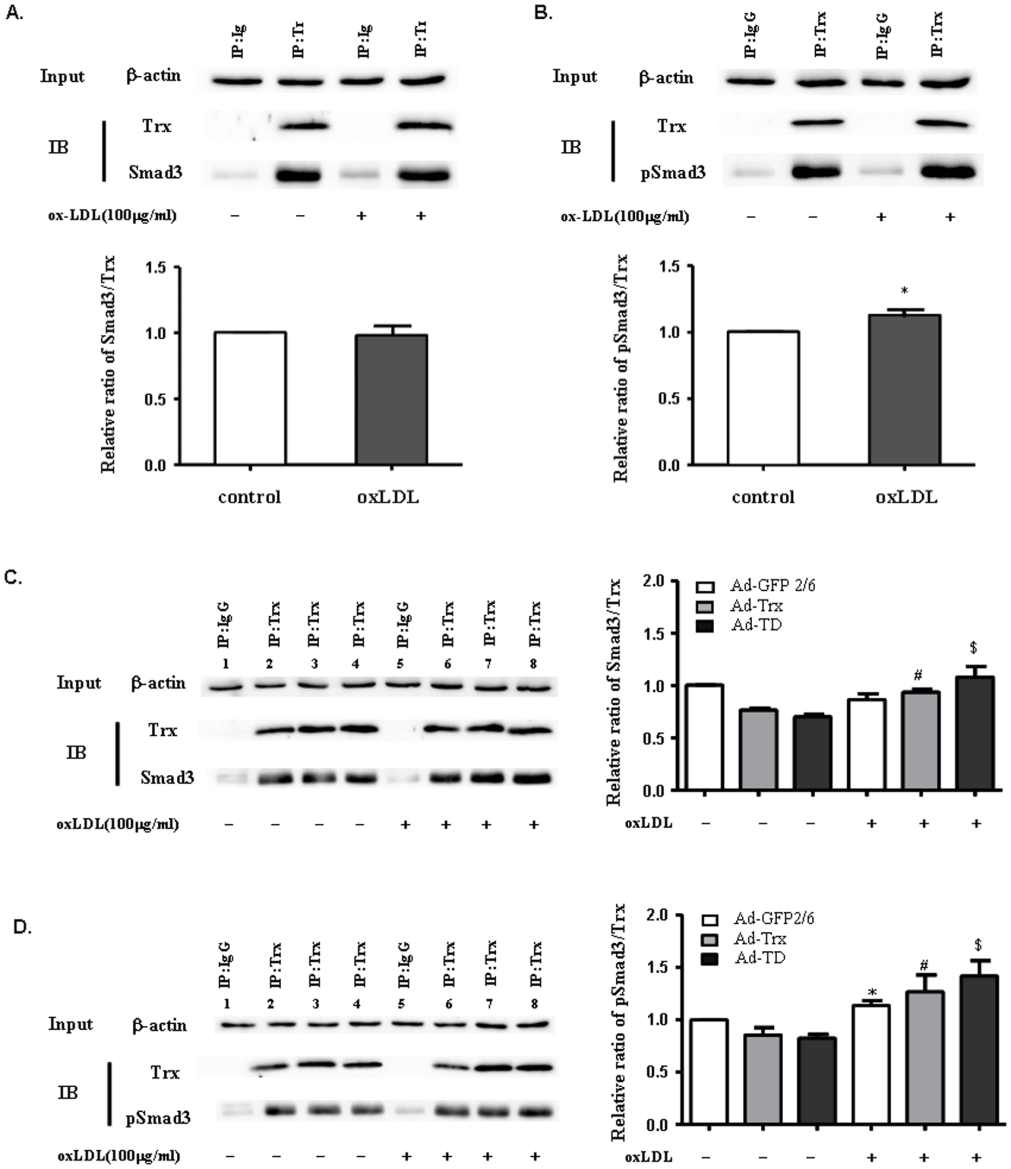
Smad3 is a newly recognized interaction partner of Trx. Reciprocal immunoprecipitation was performed using an anti-Trx antibody. Smad3 and pSmad3 were co-immunoprecipitated with Trx. Rabbit IgG served as a negative control. (**A, B**) Immunoblot with anti-Trx, anti-Smad3, or anti-pSmad3 antibody in wildtype HUVECs under basal or ox-LDL-stimulated conditions. The data from three separate experiments are expressed as mean ±SEM. *p<0.05, compared with unstimulated HUVECs. (**C, D**) Immunoblot with anti-Trx, anti-Smad3, or anti-pSmad3 antibody in Ad-GFP (1, 2, 5, 6), Ad-Trx (3, 7), and Ad-TD (4, 8) cells under basal or ox-LDL-stimulated conditions. The data from three separate experiments are expressed as mean ±SEM. *p<0.05, compared with unstimulated Ad-GFP cells; ^#^ *p*<0.05, compared with unstimulated Ad-Trx cells; ^$^ *p*<0.05, compared with unstimulated Ad-TD cells.

Co-immunoprecipitation was performed to investigate the interaction between Trx and Smad3 in HUVECs with and without ox-LDL stimulation. Polyclonal rabbit anti-Trx antibody was used to pull down the Trx interaction protein, and anti-Smad3 and pSmad3 antibodies were used to immunoblot the Trx-pull-down proteins. Normal rabbit IgG served as a negative control. As shown in Fig. 5A and B, Trx interacted with Smad3 and pSmad3 under both basal and ox-LDL-stimulated conditions. The interaction between Trx and pSmad3 was enhanced by ox-LDL stimulation. To determine whether the interactions were regulated by the Trx redox site and how the Trx redox site affected the interactions between Trx and Smad3 protein with ox-LDL stimulation, the Ad-Trx and Ad-TD groups were used to perform co-immunoprecipitation with and without ox-LDL stimulation. The data suggested that the interaction between Trx and Smad3/pSmad3 was unaffected by the Trx redox site under basal and ox-LDL stimulation conditions, ox-LDL stimulation increased the interaction between Trx1 and pSmad3/Smad3 in both the Ad-Trx and Ad-TD groups (Fig. 5C and D).

### Trx and pSmad3 had high nuclear expression in Trx-overexpressing cells

**Figure 6:**
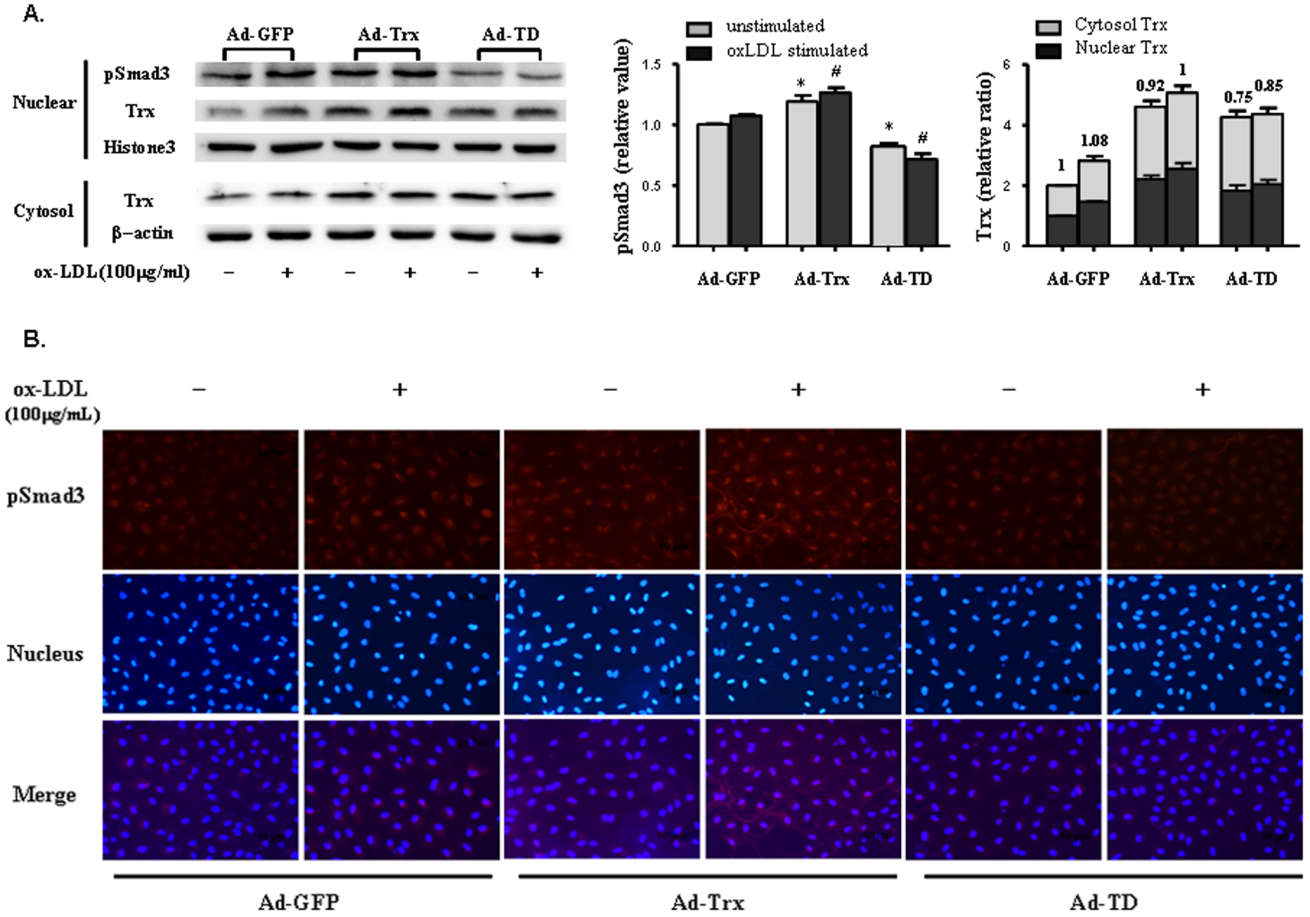
Role of Trx in nuclear translocation of pSmad3. (**A**) *Top*, Nuclear pSmad3 and Trx proteins in Ad-GFP, Ad-Trx, and Ad-TD cells. *Bottom*, Cytosolic Trx expression in Ad-GFP, Ad-Trx, and Ad-TD cells. The cells were treated with or without ox-LDL (100 µg/ml) stimulation for 6 h as indicated. *Left column*, Quantitative nuclear pSmad3 protein is shown. *Right column*, Nuclear and cytosolic Trx expression was normalized to that of the unstimulated Ad-GFP group. The numbers above the columns indicate the relative expression ratio of nuclear Trx to cytosolic Trx. The data from three separate experiments are expressed as mean ±SEM. *p<0.05, compared with unstimulated Ad-GFP cells; ^#^ *p*<0.05, compared with ox-LDL-stimulated Ad-GFP cells. (**B**) Phosphorylated Smad3 expression in Ad-GFP, Ad-Trx, and Ad-TD cells was assessed by immunofluorescent analysis.

Smad2, Smad3, and Smad4 are predominantly located in the cytoplasm. Upon TGF-β receptor activation, phosphorylated Smad2 and Smad3 translocate to the nucleus, coupled with the common mediator Smad4 [23]. Thus, the relative subcellular distribution of Smads may play an important role in TGF-β signaling and regulate Smad signaling by localizing the proteins. Trx is a stress-induced protein and translocates to the nucleus under many stimulation conditions. The aforementioned results showed that Trx is a Smad3-interacting protein, and its subcellular distribution might regulate Smad3 signaling by localizing the protein. We extracted nuclear and cytosolic proteins to analyze Trx and pSmad3 expression with and without ox-LDL stimulation. As shown in Fig. 6A, Trx and pSmad3 expression was significantly enhanced in the nucleus of Ad-Trx cells compared with Ad-GFP cells, especially with ox-LDL stimulation. Nuclear pSmad3 expression was obviously decreased in Ad-TD cells in both basal and ox-LDL-stimulated conditions. After nuclear and cytosolic Trx expression was normalized to the unstimulated Ad-GFP group, the relative expression ratios of nuclear Trx to cytosolic Trx were obtained. The data indicated that dysfunctional Trx was more likely distributed to the cytoplasm. As shown in Fig. 6B, the immunofluorescent analysis yielded results that were similar to the Western blot results. Because Trx is a Smad3-interacting protein, these results indicate that functional Trx might promote the translocation of pSmad3 to the nucleus and contribute to further activation of the Smad3 signaling pathway.

## Discussion

The present study showed that Trx inhibited the expression of the adhesion molecules VCAM-1 and ICAM-1 in HUVECs. We found that overexpression of functional Trx significantly enhanced Smad3 phosphorylation, whereas SIS3, a specific inhibitor of Smad3, reversed the Trx-induced inhibition of VCAM-1 and ICAM-1 expression after ox-LDL stimulation. These data indicate that Trx inhibited adhesion molecule expression via the Smad3 protein. Moreover, we found that Trx continued to interact with Smad3 and pSmad3, and this interaction might be responsible for the further nuclear translocation of pSmad3 in Trx-overexpressing HUVECs and activation of the Smad3 signaling pathway.

Ox-LDL is well-known to play a crucial role in the initiation and progression of atherosclerosis, which can be considered an inflammatory disease. Ox-LDL can induce proinflammatory actions in endothelial cells by increasing the expression of adhesion molecules, induction of MCP-1 production, and direct chemoattractant effect or activation of AP-1 and its transcription factors [24]. Numerous studies have reported that TGF-β has an antiatherogenic effect. TGF-β was shown to prevent the ox-LDL-induced expression of adhesion molecules [4] and contribute to plaque stabilization [5]. In endothelial cells, HDL induced TGF-β2 and activated Smad2/3 to exert its antiatherosclerotic effect [25]. Recently, Guo et al. reported that ox-LDL upregulated TGF-β1 protein production and Smad3 phosphorylation through the Ras/ERK/PLTP pathway in human alveolar type II epithelial cells [26]. However, to date, the effect of ox-LDL in the TGF-β/Smad signaling pathway in endothelial cells has not been reported. The present study found that ox-LDL reduced Smad3 expression but enhanced its phosphorylation and nuclear translocation in HUVECs.

The Trx system, including Trx, Trx reductase, and NADPH, is a ubiquitous thiol oxidoreductase system that regulates cellular reduction/oxidation (redox) status [27]. Trx reduces oxidized cysteine groups on proteins through an interaction with the redox-active center of Trx (Cys-Gly-Pro-Cys) to form a disulfide bond, which in turn can be reduced by TrxR and NADPH [9]. In the present study, wildtype Trx and redox-inactive dominant-negative mutant Trx (TD) were used to construct an adenovirus expression vector and infect HUVECs. The C32S/C35S mutant was a strong competitive inhibitor of TrxR, in which TrxR recognized the mutant with nearly equivalent affinity to Trx [28]. In contrast to the overexpression of Trx, the present data showed that TD overexpression increased ROS generation and adhesion protein expression but suppressed the Smad3 pathway by inhibiting Smad3 phosphorylation and nuclear translocation. These data indicate that inflammation related to the Smad3 pathway was regulated by the Trx redox site. Interestingly, we found that both Trx and TD promoted Smad3 phosphorylation under basal conditions, suggesting that Trx might contribute to some other unknown regulatory mechanism of Smad3 phosphorylation in addition to redox regulation.

Recent studies reported that the Smad pathway may not be a unique means by which TGF-β regulates cellular function because other signaling pathways, including the mitogen-activated protein kinase (MAPK), nuclear factor-κB, and PI3 kinase/AKT pathways, can either be induced by TGF-β or modulate the outcome of TGF-β-induced Smad signaling [29]–[32]. Smad3 contains two conserved domains, the *N*-terminal Mad homology 1 (MH1) and C-terminal Mad homology 2 (MH2) domains, and a linker domain. The MH1 domain regulates nuclear import and transcription by binding to DNA and interacting with nuclear proteins. The MH2 domain is responsible for Smad oligomerization and recognition by type I receptors and interacts with cytoplasmic adaptors and several transcription factors. The linker domain contains multiple phosphorylation sites that allow specific crosstalk with other signaling pathways, such as the ERK [33] and PKC [34] pathways, and a PY motif that mediates specific interactions with Smurfs that target Smads for degradation by the 26S proteasome [35], [36]. Trx is not only a redox protein but also an important signaling molecule. Trx is an *N*-terminal binding protein of ASK-1 and has been shown to regulate JNK/p38 MAPKs [15], [37]. Lee et al. reported that Trx bound to PTEN and reduced oxidized PTEN and may regulate the PI3K-AKT pathway through this interaction [38]. It was also reported that some subtypes of protein kinase C (PKC) interacted with Trx, which inhibits PKC activity [39]. Although no studies to date have reported that Trx regulates Smads directly, according to the literatures mentioned above[15], [29], [33], [34], [37]–[39], we hypothesized that Trx might affect Smad3 phosphorylation and degradation through the MAPK, PI3K-AKT, or PKC pathway. Based on the present results, we speculate that the interaction between Ad-Trx/TD and Smad3 protein might change the structure of Smad3 and facilitate its phosphorylation. In our study, ox-LDL stimulation induced Smad3 phosphorylation in HUVECs, and the Trx redox site affected this pathway. Thus, Trx overexpression further enhanced Smad3 phosphorylation, whereas TD overexpression downregulated Smad3 phosphorylation. Because Smad3 expression was reduced in ox-LDL-stimulated Ad-GFP and Ad-Trx cells but reversed in Ad-TD cells, ox-LDL might play a role in Smad3 degradation, which was also affected by the Trx redox site. Oxidize LDL stimulation enhanced the interaction between pSmad3 and Trx, which might promote the translocation of pSmad3 to the nucleus and contribute to further activation of the Smad3 signaling pathway. Therefore we conclude that Trx might regulate Smad3 pathway by interaction or through kinase pathway depending on its redox activity.

The present study focused on Smad3 and not Smad2. Although highly related to Smad3, Smad2 lacks the ability to bind DNA [36], [40]–[42]. Additionally, the functional properties of these two proteins may be somewhat different. The gene-targeting of Smad2 or Smad3 revealed that Smad3 cannot compensate for the defects in Smad2-null mice during early development *in vivo* and that Smad3 may play exclusive roles in immune system function [43]–[45]. For this reason, we investigated only Smad3 in the present study.

In conclusion, the present results suggested that Trx inhibited adhesion molecule expression via Smad3 protein. Although further studies are required, Trx redox was found to regulate Smad3 phosphorylation in ox-LDL-stimulated HUVECs, and Trx interacted with Smad3/pSmad3. Our study provides a potentially new molecular site for atherosclerosis prevention and treatment at the level of endothelial cells.
